# Anthocyanin, a novel and user-friendly reporter for convenient, non-destructive, low cost, directly visual selection of transgenic hairy roots in the study of rhizobia-legume symbiosis

**DOI:** 10.1186/s13007-020-00638-w

**Published:** 2020-07-06

**Authors:** Yinglun Fan, Xiuyuan Wang, Haiyun Li, Shuang Liu, Liangshen Jin, Yanyan Lyu, Mengdi Shi, Sirui Liu, Xinyue Yang, Shanhua Lyu

**Affiliations:** College of Agriculture, Liaocheng University, Liaocheng, 252000 China

**Keywords:** Reporter gene, Anthocyanin, Hairy root, Rhizobia-legume symbiosis, Soybean (*Glycine max* (L.) Merr.), *Lotus japonicus*, *Lotus corniculatus*, *Medicago truncatula*, *Rfg1*

## Abstract

**Background:**

*Agrobacterium rhizogenes*-mediated hairy root transformation provides a powerful tool for investigating the functions of plant genes involved in rhizobia-legume symbiosis. However, in the traditional identification methods of transgenic hairy roots based on reporter genes, an expensive chemical substrate or equipment is required.

**Results:**

Here, we report a novel, low cost, and robust reporter for convenient, non-destructive, and directly visual selection of transgenic hairy roots by naked eye, which can be used in the study of rhizobia-legume symbiosis. The reporter gene *AtMyb75* in *Arabidopsis*, encoding an R2R3 type MYB transcription factor, was ectopically expressed in hairy roots-mediated by *A*. *rhizogenes*, which induced purple/red colored anthocyanin accumulation in crop species like soybean (*Glycine max* (L.) Merr.) and two model legume species, *Lotus japonicas* and *Medicago truncatula*. Transgenic hairy roots of legumes containing anthocyanin can establish effective symbiosis with rhizobia. We also demonstrated the reliability of *AtMyb75* as a reporter gene by CRISPR/Cas9-targeted mutagenesis of the soybean resistance to nodulation *Rfg1* gene in the soybean PI377578 (Nod-) inoculated with *Sinorhizobium fredii* USDA193. Without exception, mature nitrogen-fixation nodules, were formed on purple transgenic hairy roots containing anthocyanin.

**Conclusions:**

Anthocyanin is a reliable, user-friendly, convenient, non-destructive, low cost, directly visual reporter for studying symbiotic nitrogen-fixing nodule development and could be widely applied in broad leguminous plants.

## Background

The leguminous plants, including crop species soybean (*Glycine max* (L.) Merr.) and model legume species *Lotus japonicus* and *Medicago truncatula*, can establish a symbiosis relationship with rhizobia and form root nodules [1]. Rhizobia reduce atmospheric dinitrogen in root nodules to ammonia that is utilized by the host legumes, resulting in improved plant growth for sustainable agriculture [2]. *Agrobacterium rhizogenes*-mediated hairy root transformation for generating composite plants composed of transgenic roots and wild-type shoot provides a powerful tool for investigating the functions of plant genes involved in legume-rhizobia symbiosis [3]. However, not all the hairy roots induced by *A*. *rhizogenes* are transgenic [3, 4]. To facilitate the identification of the transgenic roots, a reporter gene on the binary vector transformed often was employed. In the traditional screening methods of transgenic roots based on reporter genes, the β-glucuronidase (GUS) activity or fluorescent proteins (such as GFP, YFP, RFP, CFP, etc.) were the most widely utilized [1, 5–12]. GUS staining assay is destructive to plant tissues, and an expensive chemical substrate (X-Gluc) is required. Furthermore, the staining buffer includes potassium ferricyanide and potassium ferrocyanide, which are detrimental to human health [4, 13]. A prominent feature of fluorescent proteins is a non-destructive and visual reporter without a requirement of an additional substrate. However, the observation of fluorescent proteins is dependent on fluorescent microscope, and automated fluorescence background of plant tissue often interferes with screening of the transgenic roots [10]. Furthermore, eyes are subject to being sore and uncomfortable to observe fluorescence. Recently, Mitiouchkina et al. [14] reported luminescence *Nicotiana benthamiana* plants engineered by converting ceffeic acid into luciferin that is visible to the naked eye. However, it is not a convenient operation to integrate four *Neonothopanus nambi* bioluminescence genes: *nnluz* (luciferase), nnhisps (hispidin synthase), *nnh3h* (hispidin-3-hydroxylase) and *nncph* (caffeoyl pyruvate hydrolase) into plants.

Anthocyanin belongs to flavonoids that benefit for human health and is attributable to coloration of plant organs or tissues. The *Arabidopsis AtMyb75*/*PRODUCTION OF ANTHOCYANIN PIGMENTS 1* (*PAP1*, GenBank No. AY519563) encodes an R2R3 type MYB transcription factor, which regulates the production of anthocyanin [15]. To overcome the drawbacks of traditional methods for selection transgenic hairy roots, here, we developed a novel reporter gene *AtMyb75*/*PAP1* that can be used in the study of rhizobia-legume symbiosis without interfering with nitrogen-fixing nodule development. *AtMyb75*/*PAP1* was ectopically expressed in hairy roots-mediated by *A*. *rhizogenes*, that induced the purple/red colored anthocyanin accumulation in the legumes soybean (*Glycine max* (L.) Merr.) with purple hypocotyl, *L. japonicus*, *L. corniculatus*, and *M. truncatula*. Transgenic hairy roots of legumes containing anthocyanin can establish effective symbiosis with rhizobia. The *AtMyb75*/*PAP1* can be served as a reliable reporter gene was further validated by targeted editing the soybean resistance to nodulation *Rfg1* gene by CRISPR/Cas9 system in soybean PI377578 (Nod-) inoculated with *Sinorhizobium fredii* USDA193. We anticipate anthocyanin will be widely used to screen the transgenic hairy roots by *A*. *rhizogenesis*-mediated transformation in the study of rhizobia-legume symbiosis.

## Methods

### Plant materials and growth conditions

Soybean *(Glycine max* (L.) Merr.*)* PI 377578, and other different genotypes of soybean (listed in Additional file 1: Table S1) seeds used in this study were provided by the National Key Facility for Crop Gene Resources and Genetic Improvement (NFCIR), Institute of Crop Science, Chinese Academy of Agricultural Sciences. *Lotus japonicus* (*Gifu*-129), *Lotus corniculaus* L (Linn. Li’ao), and *Medicago truncatula* (accession R108) seeds were kept in our lab. Plants were cultivated in a growth chamber (24–26 °C, 16 h/8 h light/dark cycle).

### Cloning procedure of *AtMyb75*/*PAP1* and plasmids construction

**Fig. 1:**
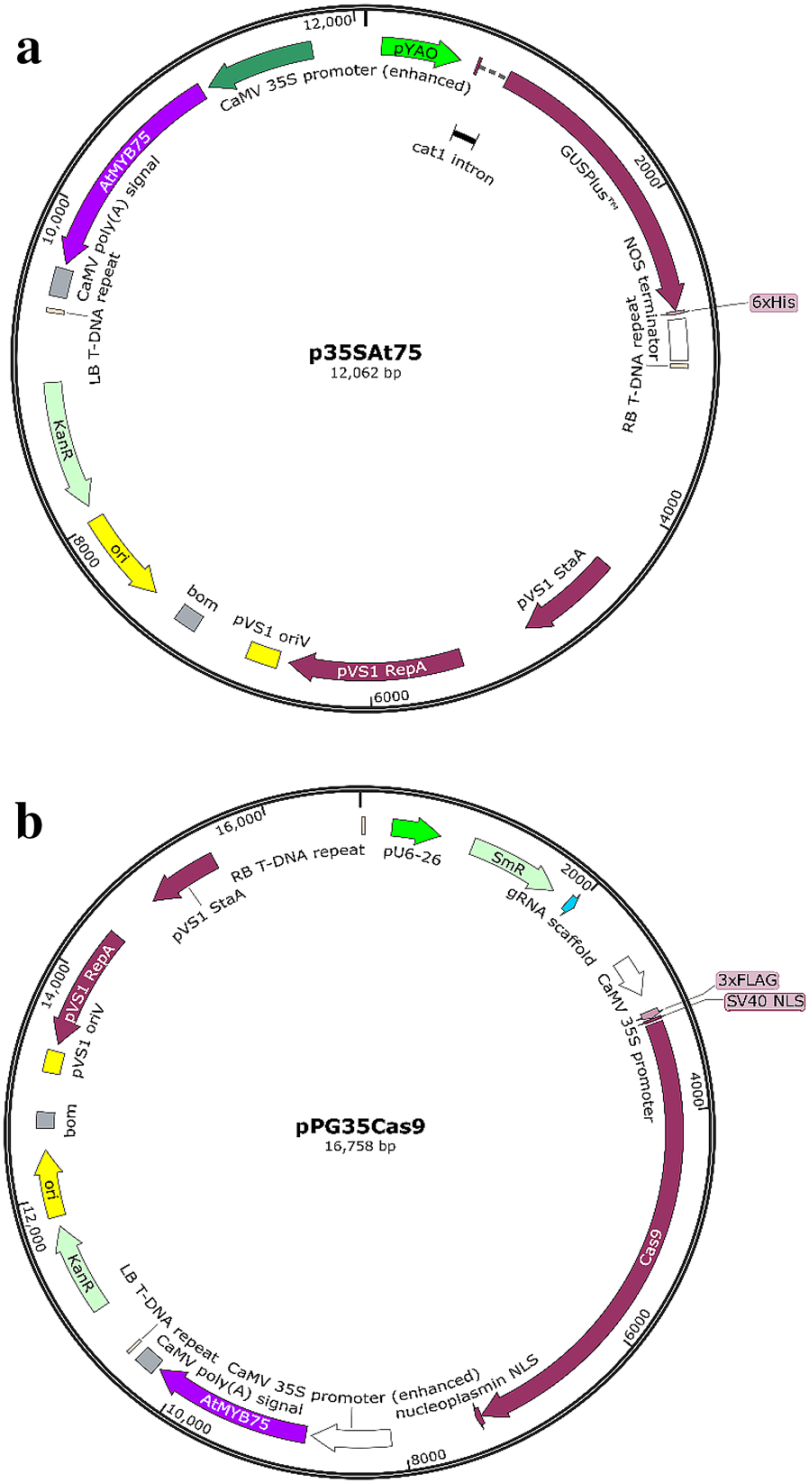
Diagrams of binary vector. **a** p35AtM75 **b** pPG35Cas9

To produce an *AtMyb75*-overexpression vector, termed as p35SAt75 (Fig. 1a), *AtMyb75* was amplified by PCR using wild-type *Arabidopsis thaliana* (Col-0) genomic DNA as a template by oligos At75SF (5′-GTATCGACTTTGTTCCATGGAGGGTTCG-3′) and At75SR (5′-ACGGTCGACCACAAACGCAAACA AATGTTCG-3′) (*Sal*I restriction site underlined). The fragment *AtMYB75* was digested with *Sal*I and then ligated into the *Xho*I restriction enzyme site of pYGUS1305 binary vector [4] containing a *GUSPlus* gene driven by *YAO* promoter [4, 16], which can be used for identification of transgenic hairy roots. In the recombinant binary vector p35SAt75, *AtMYB75* reporter gene driven by *CaMV* 35S promoter replaced the *Hpt II* (*Hygromycin Phosphotransferase II*) based on the backbone of pYGUS1305 [4]. To generate CRISPR/Cas9-mediated *Rfg1* gene knockout vector, designated as pPG35Cas9 (Fig. 1b), the DNA fragment covering *CaMV* 35S promoter, *AtMYB75* and left border of T-DNA was amplified with PCR primers At75E3 (5′-GCCAATTGATTGACAACGTTGCGTATTGGCTAGAGCAG-3′) and At75S1 (5′-AGCCGATTTTGAAACCGCGATGATCACAGGCAGCAACGCT-3′) using p35SAt75 as a template. The generated DNA fragment was then inserted in place of the *Hpt II* (*Hygromycin Phosphotransferase II*) reporter gene in the binary vector pHSE401 [17] which was linearized by *Eco*RI and *Sac*II prior to homologous recombination. One targeted site of *Rfg1* was constructed as described previously [11]. All vectors constructed were verified by sequencing. The generated binary recombinant vectors p35AtM75 and pPG35Cas9 were transformed into *A*. *rhizogenes* K599 (for soybean transformation) and ARqual strains (for *L. japonicus*, *L*. *corniculatus* and *M*. *truncatula* transformation) by electroporation, respectively.

### *A. rhizogenesis*-mediated hairy root transformation and nodulation assay

Composite soybean plants were generated using one-step *A. rhizogenes*-mediated transformation [3]. Composite *L. japonicus*, *L*. *corniculatus* [18] and *M*. *truncatula* [19] plants were generated based on previously published protocols. Nodulation assay was performed as described by Fan et al. [3] in soybean, and Okamoto et al. [18] in *L. japonicus*. Compatible rhizobia USDA110 and *microsymbiont Mesorhizobium loti MAFF303099* were used to inoculate with soybean and *L. japonicus* to induce nodulation, respectively [20, 21]. Nodulation phenotype was evaluated at 4 weeks post-inoculation. To water the composite plants, the sterile N-free nutrient solution containing 1 M CaCl_2_, 0.5 M KH_2_PO_4_, 10 mM Fe-citrate, 0.25 M MgSO_4_, 0.25 M K_2_SO_4_, 1 mM MnSO_4_, 2 mM H_3_BO_4_, 0.5 mM ZnSO_4_, 2 mM CuSO_4_, 0.1 mM CoSO_4_, 0.1 mM Na_2_MoO_4_) was used [22].

### GUS staining assay

Histochemical staining of GUS activity qualitative assay followed the protocol [13] with some modifications. The samples were incubated in a GUS staining solution (100 mM sodium phosphate at pH 7.0, 0.1% Triton X-100, 1 mg/mL X-Gluc, 1 mM potassium ferricyanide, and 1 mM potassium ferrocyanide) in the dark at 37 °C for 2-10 h. The samples stained were rinsed in a 70% ethanol for 10-20 min.

### Semi-quantitative RT-PCR

To determine whether the transformed hairy roots with anthocyanin accumulation are overexpression-*AtMYB75* or not, we carried out RT-PCR analysis. The transcripts of *AtMYB75* in the transformed soybean hairy roots were detected. To amplify *AtMYB75*, the gene-specific forward primer RTMyb75F1 (5′-TTTGTTCCATGGAGGGTTCG-3′) and reverse primer RTMyb75R1 (5′-ACCTATTCCCTAGAAGCCTATG-3′) were used and the amplification fragment covers a partial 5′ untranslated region (10-bp), a partial first exon (121-bp), the first intron (540-bp) and a partial second exon (130-bp) region. Different sizes of fragment were amplified from PCR and RT-PCR products (801-bp and 261-bp, respectively) to exclude the contamination possibility from genome DNA of the plant sample and binary vector p35AtM75 from the *A*. *rhizogenesis*. The isolation of total RNA, purification of poly (A) ± mRNA from total RNA, synthesis of first-strand cDNA were performed as previously described [23]. RT-PCR reaction was conducted according to Fan et al. [3]. The soybean *GmActin* was amplified and employed as an internal control using a forward primer GmActinF (5′-GAGCTATGAATTGCCTGATGG-3′) and a reverse primer GmActinR (5′-CGTTTCATGAATTCCAGTAGC-3′) [3].

### Statistics

Data were analyzed using Microsoft office Excel 2016 and Data Processing System (DPS) statistical software. The averages ± standard deviations of three independent experiments were calculated. Each experiment included at least 20 composite plants. Dry weight of hairy roots was measured 4 weeks post-inoculation and dried at 105 °C for 20 min, and then 80 °C for 12 h.

## Results

### Directly visual selection of transgenic hairy roots-mediated by *A*. *rhizogenes* in legumes

**Fig. 2:**
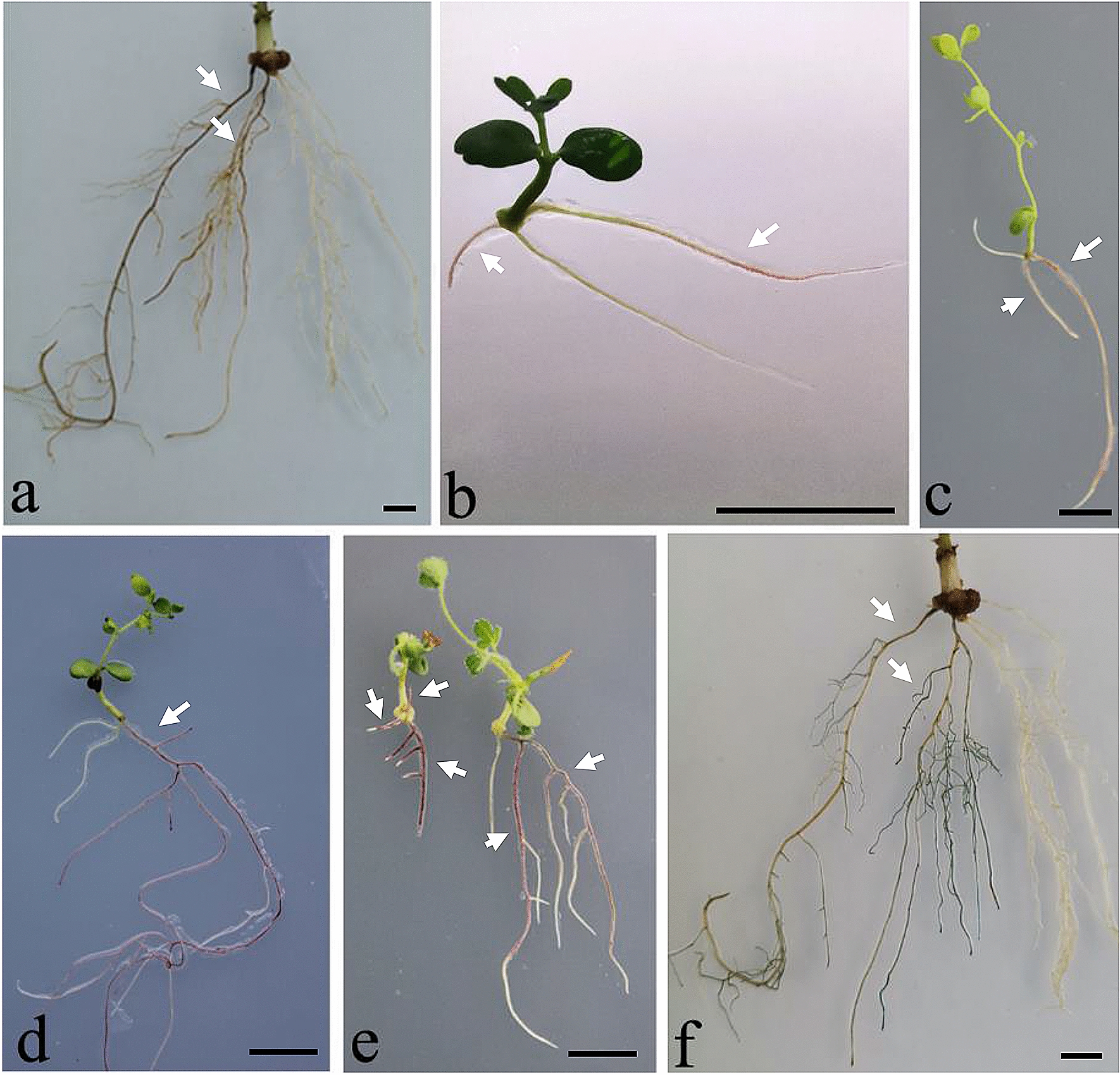
Anthocyanin accumulation in the transgenic hairy roots of leguminous plants containing 35S:: *AtMyb75*. Purple/red coloration is direct visualization in the overexpression-*AtMyb75* hairy roots of soybean PI377578 (**a**), *L. corniculaus* (**b**), *L. japonicus* (**c**, **d**) and *M. truncatula* R108 (**e**), respectively. Section **f** is the GUS stained hairy roots in section **a**, respectively. Transgene-positive roots are indicated by arrows. Bars = 1 cm

To analyze whether ectopic expression of *AtMyb75* in the roots of legumes can induce the anthocyanin accumulation, a *CaMV* 35S promoter-driven *AtMyb75* overexpression construct, p35AtM75, was transformed into the legumes soybean (*Glycine max* (L.) Merr.) PI 377578, *L. japonicus*, *L*. *corniculatus*, and *M. truncatula* (accession R108) by *A*. *rhizogenes*-mediated transformation. The ectopic expression of *AtMyb75* in the hairy roots induced purple/red colored anthocyanin accumulation in the transformed roots in soybean (Fig. 2a), *L*. *corniculatus* (Fig. 2b), *L. japonicus* (Fig. 2c, d), and *M. truncatula* (Fig. 2e). In contrast, anthocyanin was absent normally in these non-transformed roots (Fig. 2a–e). The purple/red transgenic roots with anthocyanin accumulation were directly visual to naked eyes without any exogenous substrate supplemented or equipment used, and readily distinguished from the white non-transgenic roots (Fig. 2a–e). The transgenic positive soybean roots were confirmed by GUS staining assay (Fig. 2f). Results showed that the purple/red-positive roots are consistent with the GUS-positive ones (Fig. 2a, f). Ectopic expression of *AtMyb75* in hairy roots containing anthocyanin was further confirmed by semi-quantitative RT-PCR analysis (Additional file 2: Fig. S1).

### Evaluation the reliability of anthocyanin as a reporter in the study of rhizobia-legume symbiosis

**Fig. 3:**
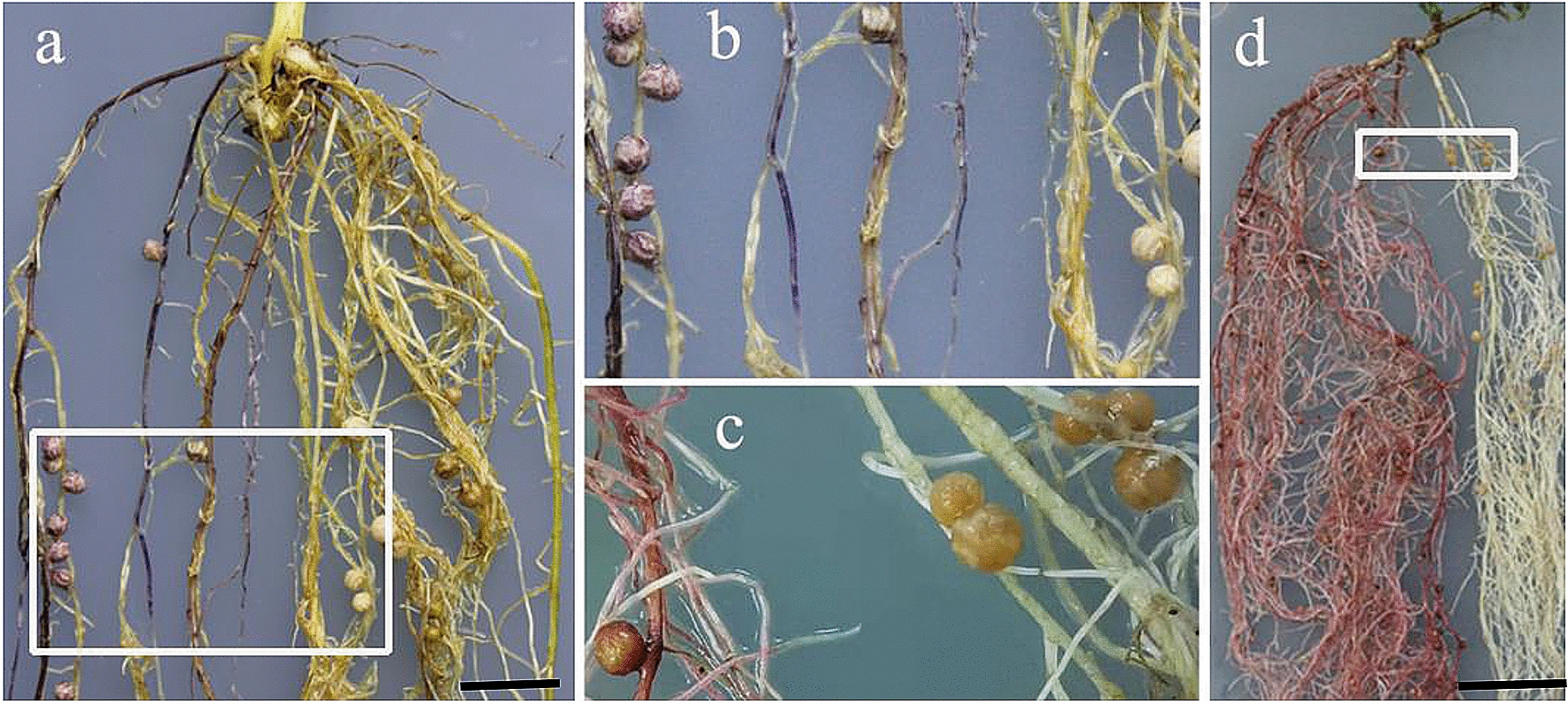
Assessment of the nodulation ability in the roots of soybean and *L. japonicas* containing anthocyanin. Mature nitrogen-fixation nodules formed on purple/red transgenic roots in soybean (**a**, **b**) and *L. japonicus* (**c**, **d**); Pictures **b** and **c** are close-up of sections **a** and **d** marked in the white box, respectively. Composite soybean plant was generated according to the protocol [22] in Fig. 4a. Bars = 1 cm

**Fig. 4:**
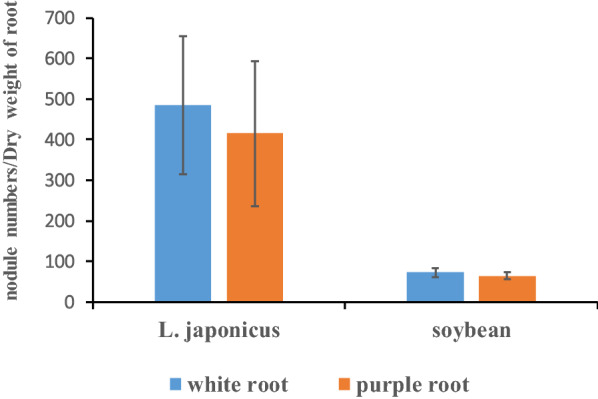
Numbers of nodules formed per dry weight of soybean and *L. japonicus* hairy roots transformed with 35S:: *AtMyb75*

To evaluate whether the produced anthocyanin in transgenic roots interferes with legume nodulation and can be served as a reporter applied for nitrogen-fixing nodule development, compatible rhizobia USDA110 and *M. loti MAFF303099* were used to inoculate with soybean and *L. japonicus* to induce nodulation, respectively. Mature nitrogen-fixing nodules were formed on purple/red transgenic roots produced anthocyanin in soybean (Fig. 3a, b) and *L. japonicus* (Fig. 3c, d). Purple/red anthocyanin was visual to naked eyes in both transgenic roots and nodules (Fig. 3). The result indicated that transgenic roots of legumes with anthocyanin accumulation nodulated normally and could establish effective symbiosis with rhizobia. The nodule numbers per root dry weight of transgenic roots were not significantly different (p ˂ 0.05) from that of non-transgenic roots, even though the transgenic roots with a higher level of anthocyanin accumulation compared with non-transgenic roots (infected with K599 harboring pCAMBIA1305.1) were used for statistical analysis (Fig. 4). These results indicated that anthocyanin do not affect the interaction of the plant roots with rhizobia in soybean and *L. japonicus*.

**Fig. 5:**
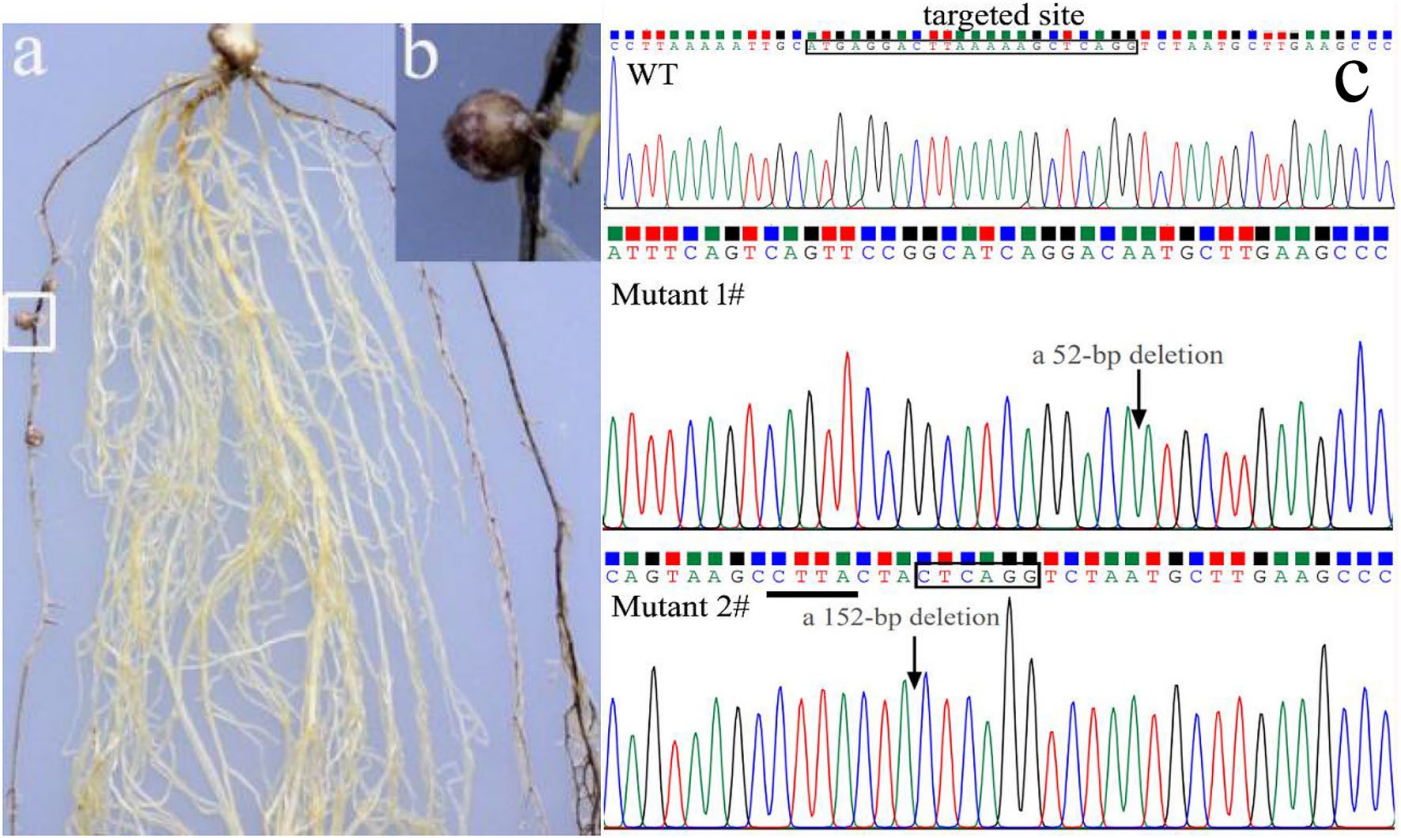
CRISPR/Cas9-mediated knockout of *Rfg1* in the soybean PI377578 (Nod-) background inoculated with USDA193. **a** Mature nodules formed on overexpression-*AtMyb75* hairy roots. Close-up of section **a** marked in the white box was shown in section **b**. **c** A sequencing identification from CRISPR/Cas9-mediated knockout of *Rfg1* in the soybean PI 377578 (Nod-) background. An example of sequencing analysis of the DNA from wild type nodule (WT) and targeted site and PAM site (AGG) are shown within the box. Transgenic nodules revealed that two mutant alleles with a 52-bp (mutant 1#) and 152-bp (mutant 2#) deletion were caused, respectively. The deleted site is shown by a black arrow. Bars = 1 cm

Specific rhizobia can establish effective symbiosis with specific legumes species (or genotypes of legumes) and therefore form nitrogen-fixation nodules [11]. For instance, soybean PI377578 prevents *Sinorhizobium fredii* USDA193 from nodulation (Nod-) and the dominant *Rfg1* in the soybean is responsible for nodulation restriction (our unpublished data). To further test the reliability of *AtMyb75* as a reporter gene, *Rfg1* was knockout by the CRISPR/Cas9 system in PI377578 inoculated with USDA193. Transgenic roots can form nodules only containing homozygous or bi-allelic *Rfg1* mutation [11]. Mature nitrogen-fixation nodules, as expected, were successfully formed on purple/red transgenic roots produced anthocyanin (Fig. 5a, b). No nodules were formed on non-transgenic roots (Fig. 5a). The transgenic nodules were confirmed by PCR analysis and sequencing (Fig. 5c).

### Application of the anthocyanin as a reporter to different genotypes of soybean

To assess whether overexpression-*AtMyb75* in hairy roots can induce anthocyanin accumulation in various soybean cultivars, we analyzed 86 genotypes of soybean. Visual anthocyanin was accumulated in 39 out of 86 surveyed soybean varieties. Overexpression-*AtMyb75* transgenic roots in soybean cultivars with purple hypocotyl could be accumulated purple/red anthocyanin. Compared with this, there is no purple/red anthocyanin accumulation in overexpression-*AtMyb75* transgenic roots induced from the soybeans with green hypocotyl (Additional file 1: Table S1). The anthocyanin accumulation in overexpression-*AtMyb75* roots might be linked with purple hypocotyl in soybean. This requires further experimental testing of more different genotypes of soybean with purple and green hypocotyls.

## Discussion

In this study, whether anthocyanin could be taken as a novel reporter in studying legume-rhizobia symbiosis was analyzed. Ectopic expression of *AtMyb75* in soybean with purple hypocotyl, leguminous plant *L. japonicas*, *L. corniculaus* and *M. truncatula* resulted in purple/red anthocyanin production in transgenic hairy roots-mediated by *A*. *rhizogenes* transformation. Transgenic hairy roots were easily to be distinguished from non-transgenic roots under white light by the color conferred by the anthocyanin. The transgenic soybean and *L. japonicus* hairy roots containing 35S:: *AtMyb75* which was accumulated with anthocyanin can form mature nodules inoculated with compatible rhizobia. The targeted mutation of *Rfg1* in soybean PI377578 by CRISPR–Cas9 system further validated that anthocyanin served as a reporter is reliable. Taking these findings together, we conclude that anthocyanin can be served as a novel reporter applied in directly visual selection transgenic hairy roots-mediated by *A*. *rhizogenes* transformation in the study of rhizobia-legume symbiosis.

Anthocyanin can be synthesized in the early hairy roots with ~ 1 cm length (Fig. 2b, c, e) and persisted through later stage of nodule development (Fig. 3). These results indicated that anthocyanin provides an early-stage selection tool for the transgenic hairy roots. During preparing this paper, another study also demonstrated that the application of anthocyanin as a reporter in *M*. *truncatula* for studying nitrogen-fixing nodule development [24], which corroborates the reliability of our method. Based on our results and previous study, we conclude that anthocyanin is a user-friendly, convenient, non-destructive, low cost, directly visual ideal reporter in research on rhizobia-legume symbiosis.

In this study, we also observed that some genotypes of soybean with green hypocotyl containing overexpression-*AtMyb75* can not induce the production of anthocyanin in hairy roots. This might result from no downstream or interaction genes regulated by *AtMyb75* involving in synthesis of anthocyanin in the roots of these genotypes. However, anthocyanin can be synthesized by transformed with overexpression-*AtMyb75* in the soybeans with purple hypocotyl tested. Anyway, till this study, anthocyanin served as a reporter can be applied in some leguminous plant species. In the future, with the elucidating of the synthesis mechanism of anthocyanin, if possible, anthocyanin can be accumulated by regulating the expression of other gene(s) involving in the synthesis of anthocyanin in those soybeans with green hypocotyl and therefore widely applied in all genotypes of soybean as a reporter.

## Conclusions

Anthocyanin is a user-friendly, healthy to human and environment, convenient, non-destructive, low cost, directly visual reporter gene and suitable for transgenic selection of hairy roots-mediated by *A*. *rhizogenes* transformation in studying the nitrogen-fixation nodules development.

## Supplementary information

**Additional file 1: Table S1.** Anthocyanin accumulation on transgenic hairy roots containing 35S:: *AtMyb75* in various soybean cultivars. Data were obtained by three independent experiment with at least infected 8 seedlings.

**Additional file 2: Fig. S1.** Transcription analysis of *AtMyb75* in induced soybean hairy roots by RT-PCR. RNAs were extracted from independent hairy roots induced by *A*. *rhizogenes* carrying p35AtM75 construct. *Gm*-*Actin* (27 cycles) (**a**) and *AtMyb75* (30 cycles) (**b**) were amplified, respectively. Lane 1–4, non-transgenic white root inoculated with K599-p35AtM75; lane 5, p35AtM75 plasmid; lane 6–11, independent transgenic root with anthocyanin accumulation inoculated with K599-p35AtM75; lane 12, K599; lane 13, ddH_2_O. M, DL2000 DNA ladder bought from Sangon Biotech (band is 100 bp, 250 bp, 500 bp, 750 bp, 1000 bp, 2000 bp, respectively).

## Data Availability

All data supporting the conclusions of this article are included in this article.

## Abbreviation

CRISPR/Cas9: clustered regularly interspaced short palindromic repeats-associated Cas9
GFP/RFP/YFP/CFP: Green/Red/Yellow/Cyan fluorescent protein
GUS: β-glucuronidase
RT-PCR: reverse transcriptase polymerase chain reaction
bp: base pairs
K599-p35AtM75: K599 harboring p35AtM75 vector
