# Supplementary figures and images for: Anthocyanin, a novel and user-friendly reporter for convenient, non-destructive, low cost, directly visual selection of transgenic hairy roots in the study of rhizobia-legume symbiosis
Source: Plant Methods. 2020 Jul 6;16:94. doi: 10.1186/s13007-020-00638-w (PMC7339386; doi:10.1186/s13007-020-00638-w)

Fig. S1


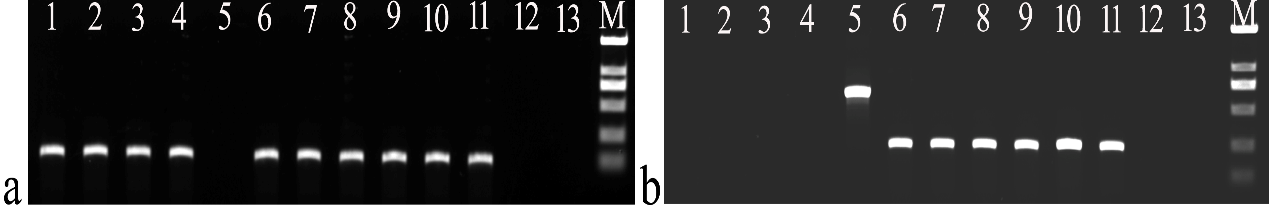

Supplement: Supplementary file 2 — Additional file 2: Fig. S1. Transcription analysis of AtMyb75 in induced soybean hairy roots by RT-PCR. RNAs were extracted from independent hairy roots induced by A. rhizogenes carrying p35AtM75 construct. Gm-Actin (27 cycles) (a) and AtMyb75 (30 cycles) (b) were amplified, respectively. Lane 1–4, non-transgenic white root inoculated with K599-p35AtM75; lane 5, p35AtM75 plasmid; lane 6–11, independent transgenic root with anthocyanin accumulation inoculated with K599-p35AtM75; lane 12, K599; lane 13, ddH2O. M, DL2000 DNA ladder bought from Sangon Biotech (band is 100 bp, 250 bp, 500 bp, 750 bp, 1000 bp, 2000 bp, respectively). [file 13007_2020_638_MOESM2_ESM.docx]
